# YOLO-SUMAS: Improved Printed Circuit Board Defect Detection and Identification Research Based on YOLOv8

**DOI:** 10.3390/mi16050509

**Published:** 2025-04-27

**Authors:** Ying Tang, Runhao Liu, Sheng Wang

**Affiliations:** 1 School of Mechanical and Electrical Engineering, Chengdu University of Technology, Chengdu 610059, China; tangying@mail.cdut.edu.cn; 2 School of Computer and Network Security, Chengdu University of Technology, Chengdu 610059, China; 18282930584@163.com

**Keywords:** defect detection, YOLOv8n, PCB, deep learning, multi-scale feature fusion

## Abstract

Aiming at the demand for defect detection accuracy and efficiency under the trend of high-density and integration in printed circuit board (PCB) manufacturing, this paper proposes an improved YOLOv8n model (YOLO-SUMAS), which enhances detection performance through multi-module collaborative optimization. The model introduces the SCSA attention mechanism, which improves the feature expression capability through spatial and channel synergistic attention; adopts the Unified-IoU loss function, combined with the dynamic bounding box scaling and bi-directional weight allocation strategy, to optimize the accuracy of high-quality target localization; integrates the MobileNetV4 lightweight architecture and its MobileMQA attention module, which reduces the computational complexity and improves the inference speed; and combines ASF-SDI Neck structure with weighted bi-directional feature pyramid and multi-level semantic detail fusion to strengthen small target detection capability. The experiments are based on public datasets, and the results show that the improved model achieves 98.8% precision and 99.2% recall, and mAP@50 reached 99.1%, significantly better than the original YOLOv8n and other mainstream models. YOLO-SUMAS provides a highly efficient industrial-grade PCB defect detection solution by considering high precision and real-time performance while maintaining lightweight characteristics.

## 1. Introduction

PCB defect detection is a critical step in ensuring the quality and reliability of PCBs [1]. As the manufacturing requirements for PCBs are evolving in the direction of high density and integration [2], it is of great significance to study efficient methods for achieving low-cost defect detection. Traditional PCB manual inspection is a time-consuming, laborious, and inefficient method [3], which cannot meet the basic requirements of modern production, thus giving rise to electrical inspection, automatic ray detection, thermal imaging inspection [4,5,6], and other inspection means. Although these means can meet the needs of PCB defect detection, they have their limitations, such as electrical inspection, which may cause potential defects, while radiographic and thermographic inspection require expensive equipment to obtain defect information.

In recent years, deep learning has become increasingly mature during target inspection. Deep learning-based target detection algorithms are categorized into single-stage and two-stage detection algorithms [7]. Two-stage detection algorithms are mainly represented by the SPP-Net model, FastRCNN, and Faster-RCNN algorithms [8,9,10], but there are problems such as a complex training process, slow detection speed, and inability to perform real-time detection, which does not apply to industrial PCB defect detection. Single-stage algorithms are mainly represented by YOLOv2, YOLOv3, YOLOv4, YOLOv5, YOLOv6, YOLOv7, and SSD network structure [11,12,13,14,15,16], which have the advantages of fast detection speed and less computation and can satisfy the basic needs of industrial detection.

Tang et al. [17] proposed an online PCB detection algorithm in which GPP (Group Pyramid Pooling Module) can efficiently use features at different scales. Li et al. [18] introduced an extended feature pyramid network detection model that efficiently combines high-level semantic details with low-level geometrical features and introduced a focus loss function. The model exhibits strong transferability; however, in an actual industrial production environment, the algorithm’s accuracy is still low, and the generalization ability is poor due to problems such as noise interference and uneven exposure, solidifying it as unsuitable for industrial production applications. Ding et al. [19] constructed a TDD-Net using the FasterR-CNN framework, which employs k-means clustering to design suitable anchor points, and TDD-Net enhances the relationship between different levels of feature maps and utilizes the underlying structural information, making it particularly ideal for detecting tiny defects. Wu et al. [20] introduced a lightweight network model, which is an improvement of YOLOv5, and adopts the GhostConv and GhostBottleneck methods to achieve a more lightweight algorithmic structure, which significantly reduces the number of parameters and computational complexity (Flops) required by the model, and integrates a dual-attention mechanism, combining the SE structure and CBAM, to enhance the algorithmic performance and effectively solve the difficulties of tiny defect detection. In addition, Tsai and Chou proposed a fast PCB-accurate localization method based on deep neural network regression, significantly improving the efficiency and accuracy of PCB defect detection [21]. In terms of the attention mechanism, Chen et al. proposed a transformer-YOLO-based PCB defect detection method, which significantly improves the model’s ability to detect small target defects by introducing channel attention and spatial attention mechanisms [22]. Tang et al. proposed an improved YOLOv5 algorithm for PCB surface defect detection, which improves the detection performance of the model by introducing a lightweight feature fusion network [23]. Annaby et al. proposed an improved low-complexity NCC method to locate missing ICs on PCBs by converting 2D sub-images to 1D feature descriptors and performing computations in the Transform domain, which results in improved speed and robustness to noise [24]. Bhattacharya et al. introduced an end-to-end deep learning model that uses an improved YOLOv5 model in combination with transformers to quickly and accurately detect and classify manufacturing defects on PCBs, greatly reducing the number of parameters in the model while achieving superior performance [25]. In addition, there are other studies to improve YOLO small object detection, such as YOLO-Z (reference [26]), which enhances small object detection by improving the feature pyramid, but relies on static channel attention (such as SE module) and does not solve the problem of spatial-channel information fragmentation. Transformer-YOLO (reference [27]) uses self-attention to capture global relationships, but its high computational complexity makes it difficult to adapt to real-time detection. The attention mechanism mentioned in this study dynamically adjusts the number of channel groups through the progressive channel compression strategy to reduce redundant calculations, and strengthens the response of small target features through bidirectional weight allocation, which is superior to the traditional attention in focusing the missing solder joint.

The rapid development of deep learning technology has provided new solutions in the field of PCB defect detection [28], and the use of deep learning for PCB defect detection has become a mainstream trend. In this study, we explored an improved deep-learning model to further enhance the accuracy and efficiency of PCB defect detection. To further enhance the detection of various PCB defects, this study proposes a more accurate and lightweight model based on the YOLOv8n network. The main innovations and improvements introduced in the approach include the following:

1. Adding the SCSA attention mechanism enhances the generalization ability in visual tasks such as image classification, target detection, and semantic segmentation.
2. Introducing Unified-IoU loss function, optimized for high-quality targets. Unified-IoU introduces the FocalBox method, which allocates weights by scaling the prediction frames with the real frames and employs an annealing strategy (introducing the dynamic parameter epoch), which gradually shifts the model’s attention from the low-quality prediction frames to the high-quality prediction frames, balancing the training speed and detection accuracy.
3. The backbone part combines the Universal Inverted Bottleneck (UIB) module of MobileNetV4 to form the C2fUIB model, which improves the performance of the lightweight network, and the MobileMQA attention mechanism further optimizes the inference speed of the mobile gas pedal.
4. The neck part combines the novel network structure of SDI and ASF-YOLO to enhance the performance of target detection and segmentation tasks. The SDI module and the weighted bidirectional feature pyramid network of ASF-YOLO enhance the semantic and detailed information of the image to achieve excellent performance in instance segmentation.
5. The effectiveness of each part of this paper and the performance advantages of the proposed algorithm are verified by image enhancement, ablation experiments, and comparison experiments on the PKU-MARKET-PCB public dataset.

## 2. Methodology

### 2.1. Overall Architecture of YOLO-SUMAS

**Figure 1:**
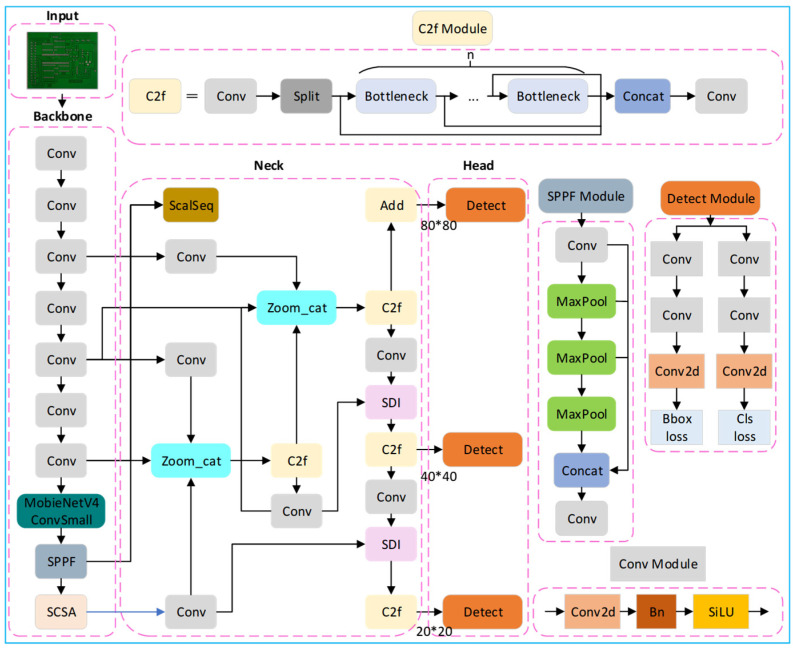
Diagram of the improved network structure.

Due to the small size of the inspection target of PCB circuit boards and the limited number of data sets, targeted improvement optimization is needed according to the feature distribution of the sample data when using the general YOLOv8 model for defect detection. According to the depth and width of the network, YOLOv8 can be categorized into five versions: YOLOv8n, YOLOv8s, YOLOv8m, YOLOv8l, and YOLOv8x. Among them, YOLOv8n, with a smaller number of parameters and good computational efficiency, is chosen as the baseline model for this study. Our design motivation is to address the three major challenges of PCB high-density, small-target defects: noise interference, positioning bias, and computational efficiency. On this basis, the images were input, the SCSA attention mechanism and MobileNetV4 neural network architecture were introduced into the backbone network part, the Unified-IoU loss function was used, and the ASF-SDI network structure was added to the neck part; adjusting the model structure, finally, it can effectively detect various defects in the PCB image, including short circuit, open circuit, spurious copper, and so on—the improved network structure diagram is shown in Figure 1. The modules work together: SCSA enhanced the image feature expression, MobileNetV4 performed lightweight reasoning for the model, ASF-SDI realized multi-scale fusion, and Unified-IoU optimized positioning.

### 2.2. Key Technical Innovations

#### 2.2.1. SCSA Attention Mechanism [29]

**Figure 2:**
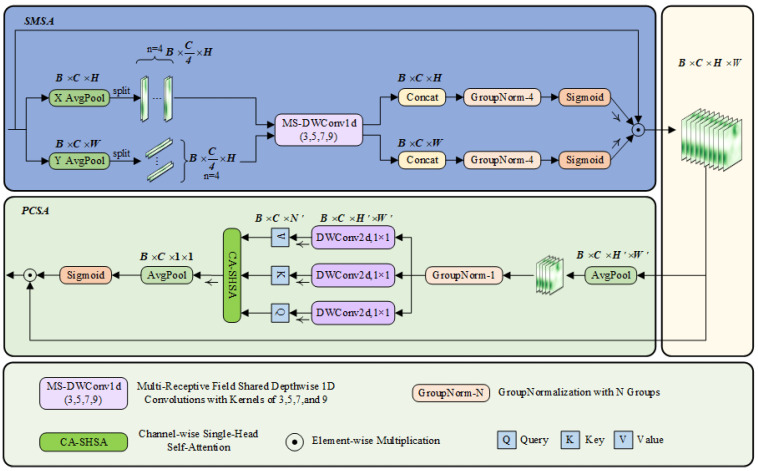
Framework diagram of the SCSA attention mechanism.

To solve the problem of feature dilution of traditional attention (such as CBAM) on PCB small targets, the SCSA attention mechanism is introduced. SCSA (Spatial and Channel Synergistic Attention) is a novel attention mechanism designed to enhance feature extraction in visual tasks by synergizing spatial and channel dimensions. SCSA consists of two main modules: shared multisemantic spatial attention (SMSA) and progressive channel self-attention (PCSA). Its framework is shown in Figure 2, where B denotes the batch size, C represents the number of channels, and H and W correspond to the height and width of the feature mapping, respectively. The variable n indicates the number of groups into which the sub-features are divided.

The SMSA module extracts spatial information at different semantic levels from multiple independent sub-features using a 1D convolution with multi-scale depth sharing. The input feature map $X\in R^{B\times C\times H\times W}$ is decomposed into two one-dimensional sequences in the height and width directions, $X_{H}$ and $X_{W}$, and then normalized by GroupNormalization (GN) to generate the spatial attention graph. The main calculation formula is as follows:

(1) $${\tilde{X}}_{H}^{i}={DWConvld}_{k_{i}}^{\frac{C}{K}\to \frac{C}{K}}\left(X_{H}^{i}\right)$$

(2) $${\tilde{X}}_{W}^{i}={DWConvld}_{k_{i}}^{\frac{C}{K}\to \frac{C}{K}}\left(X_{W}^{i}\right)$$

${\tilde{X}}^{i}$ denotes the spatial structure information of the *i*th sub-feature obtained after the lightweight convolution operation. $K_{i}$ denotes the convolution kernel applied to the *i*th sub-feature.

(3) $${\mathrm{Attn}}_{H}=\sigma \left({\mathrm{GN}}_{H}^{K}\left(\mathrm{Concat}\left({\tilde{X}}_{H}^{1},{\tilde{X}}_{H}^{2},\ldots ,{\tilde{X}}_{H}^{K}\right)\right)\right)$$

(4) $${\mathrm{Attn}}_{W}=\sigma \left({\mathrm{GN}}_{W}^{K}\left(\mathrm{Concat}\left({\tilde{X}}_{W}^{1},{\tilde{X}}_{W}^{2},\ldots ,{\tilde{X}}_{W}^{K}\right)\right)\right)$$

(5) $$SMSA\left(X\right)=X_{S}={Attn}_{H}\times {Attn}_{W}\times X$$

σ(·) means normalized Sigmoid, and ${GN}_{H}^{K}(\cdot )$ and ${GN}_{W}^{K}(\cdot )$ means having *K* along the *H* and *W* dimensions, respectively Group GN.

The PCSA module optimizes the channel features using a progressive compression strategy and a channel self-attention mechanism. The input features are pooled and compressed, and the single-head self-attention mechanism calculates the similarity between channels. The main computational formula is as follows:

(6) $$X_{P}={Pool}_{\left(7,7\right)}^{\left(H,W\right)\to \left(H^{\prime },W^{\prime }\right)}\left(X_{S}\right)$$

(7) $$F_{proj}={DWConvld}_{\left(1,1\right)}^{C\to C}$$

(8) $$Q=F_{proj}^{Q}\left(X_{P}\right),K=F_{proj}^{K}\left(X_{P}\right),V=F_{proj}^{V}\left(X_{P}\right)$$

(9) $$X_{attn}=Attn\left(Q,K,V\right)=Softmax\left(\frac{{QK}^{T}}{\sqrt{C}}\right)V$$

(10) $$PCSA\left(X_{S}\right)=X_{C}=X_{S}\times \sigma \left({Pool}_{\left(H^{\prime },W^{\prime }\right)}^{\left(H^{\prime },W^{\prime }\right)\to \left(1,1\right)}\left(X_{attn}\right)\right)$$

${Pool}_{\left(k,k\right)}^{\left(H,W\right)\to \left(H^{\prime },W^{\prime }\right)}(\cdot )$ denotes a pooling operation with kernel size *k* × *k* for (*k*, *k*), which rescales the resolution from (*H*, *W*) to (*H*’, *W*’). $F_{proj}(\cdot )$ represents the linear projection that generates the query, key, and value.

*SCSA* realizes the synergistic effect of spatial and channel attention by connecting the *SMSA* and *PCSA* modules in tandem. *SMSA* provides accurate spatial a priori information, while *PCSA* mitigates multi-semantic discrepancies and facilitates feature fusion through the self-attention mechanism. Ultimately, the output of *SCSA* is

(11) $$SCSA\left(X\right)=PCSA\left(SMSA\left(X\right)\right)$$

#### 2.2.2. Loss Function [30]

To solve the gradient imbalance problem of traditional IoU in dense defects, we introduce Unified-IoU loss function. Unified-IoU balances the model convergence speed and high-precision detection performance by dynamically adjusting the scaling ratio of the bounding box and the weight allocation strategy. The core innovations of Unified-IoU are as follows.

First, we incorporate a dynamic bounding box scaling mechanism. Dynamically adjust the IoU loss weights by scaling the dimensions of the prediction box and the real box. Scaling down the bounding box reduces the IoU value of the high-quality prediction box and increases its loss weight, prompting the model to focus on high-precision detection; scaling up the bounding box reduces the loss weight of the low-quality prediction box, accelerating model convergence. The scaling ratio is controlled by the dynamic hyperparameter “*ratio*”, and its adjustment strategies include linear, cosine, and fractional decay.

Linear decay:

(12) ratio=−0.005×epoch+2

Cosine decay:

(13) $$ratio=0.75\times \cos\left(\frac{\pi \times epoch}{300}\right)+1.25$$

Second, we incorporate the Dual-attention mechanism. Combine the FocalLoss idea to design a bidirectional weight assignment, assigning higher weight to the low-confidence prediction frame (focusing on the difficult samples) and extra weight to the high-IoU prediction frame (improving the performance of dense scene detection). The final loss function incorporates geometric metrics and dynamic weights:

(14) $${\mathrm{UIoU}}_{\mathrm{loss}}=\left(1-p\right)\times {\mathrm{IoU}}_{\mathrm{loss}}$$

(15) $${\;IoU}_{loss}=1-\frac{P\cap P_{gt}}{P\cup P_{gt}}$$

where *p* is the confidence of the predicted box, and ${\mathrm{IoU}}_{\mathrm{loss}}$ is the original IoUloss. *P* is the predicted box, and $p_{\mathrm{gt}}$ is the Ground Truth. Unified IoU solves the problem of the traditional IoU loss in which low-quality samples dominate the gradient and achieves a balance between high accuracy and high efficiency.

#### 2.2.3. Neural Network Architecture [31]

To solve the problem of insufficient inference speed of YOLOv8n’s C2f structure on mobile terminals, we add the MobileNetV4 module, a highly optimized neural network architecture. It employs the Universal Inverted Bottleneck (UIB) and the MobileMQA attention module optimized for mobile gas pedals to improve inference speed and computational efficiency without sacrificing accuracy.

**Figure 3:**
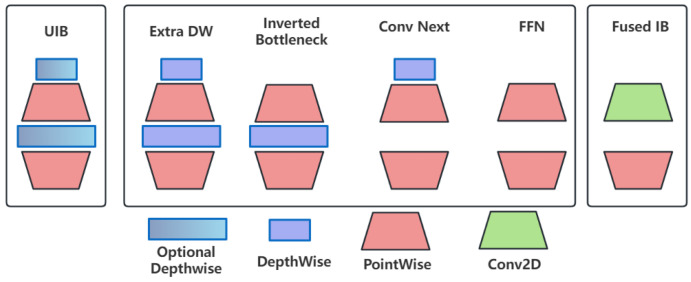
Generic reverse bottleneck module.

The Universal Inverted Bottleneck (UIB) module of MobileNetV4 forms a C2fUIB model with YOLOv8, as shown in Figure 3. The UIB combines elements from MobileNetV2, ConvNext, and ViT, as well as the feedforward network in the ConvNext block and the Visual Transformer (ViT), among other things, to improve the lightweight performance of the network. The architecture allows for the adaptive and efficient scaling of the model to various platforms without over-complicating the architecture search process.

In addition, MobileNetV4 includes a novel attention mechanism called *MobileMQA*, which significantly improves the speed of reasoning on mobile gas pedals by optimizing the ratio of arithmetic operations to memory accesses. The computation process of *MobileMQA* is as follows:

(16) $$Mobil\_MQA(X)=Concat\left({attention}_{1},\ldots ,{attention}_{n}\right)W^{O}$$

(17) $${attention}_{j}=softmax\left(\frac{\left({XW}^{Q_{j}}\right){\left(SR\left(X\right)W^{K}\right)}^{T}}{\sqrt{d_{k}}}\right)\left(SR\left(X\right)W^{V}\right)$$

Among them, *SR*—spatial downsampling operation—can be a 3 × 3 depth-wise separable convolution with step size two. $W^{Q},W^{K},W^{V}$ are Query, Key, and Value weight matrices. $d_{K}$ is the dimension of the key vector.

In addition to the above two modules, MobileNetV4 introduces an improved NAS policy that increases search efficiency and improves model quality through a combination of coarse- and fine-grained search. With this approach, MobileNetV4 can achieve Pareto-optimized performance in most cases and an optimal balance of efficiency and accuracy on most devices. To make the model generally efficient, various bottlenecks limit the performance of the model, and these bottlenecks depend heavily on the peak computational throughput and peak memory bandwidth of the hardware, so we use the Roofline model, which estimates the performance of a given workload and predicts whether it will be a memory bottleneck or a computational bottleneck. The Roofline model is computed using the formula

(18) $$ModelTime=\sum_{i}\max\left({MACTime}_{i},{MemTime}_{i}\right)$$

(19) $${MACTime}_{i}=\frac{{LayerMACs}_{i}}{{PeakMAC}_{s}}$$

(20) $${MemTime}_{i}=\frac{{WeightBytes}_{i}+{ActivationBytes}_{i}}{PeakMemBW}$$

*LayerMACs* is the multiply-accumulate operand at layer *i*. *PeakMACs* is the peak computational throughput of the hardware. *WeightBytes* and *ActivationBytes* are the number of memory accesses for the weight and activation. *PeakMemBW* is the peak memory bandwidth of the hardware.

#### 2.2.4. Neck Network Architecture

To solve the loss of details in PCB by traditional FPN, a new Neck network architecture is formed by utilizing a multi-level feature fusion module (SDI) paired with the classical weighted bidirectional feature pyramid network ASF-YOLO, where the SDI module enhances semantic and detail information in the image by integrating the hierarchical feature maps generated by the encoder. The model improves the accuracy and speed of processing images by combining spatial and scale features.

ASF-YOLO (AttentionalScaleSequenceFusionbasedYOLO) [32] is a new instance segmentation model based on the YOLO framework, which aims to improve the accuracy and speed of image segmentation by fusing spatial and scale features. The model introduces the following two key modules based on the YOLO segmentation framework.

1. Triple Feature Encoder (TFE) module

**Figure 4:**
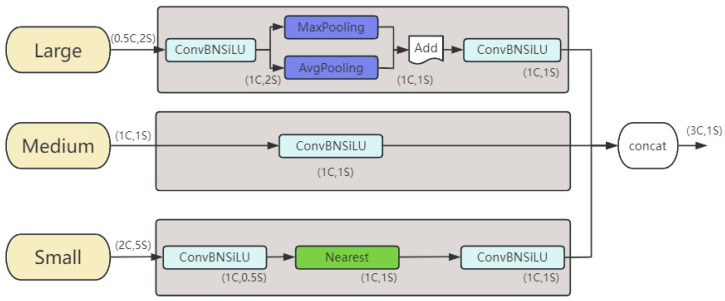
Structure of TFE module.

It increases the detailed information of small objects by fusing feature maps of different scales, as shown in Figure 4, where C represents the number of channels and S represents the feature map size. Each triple feature encoder module uses three feature maps of different sizes as input.

2. Channel and Position Attention Mechanism (CPAM)

**Figure 5:**
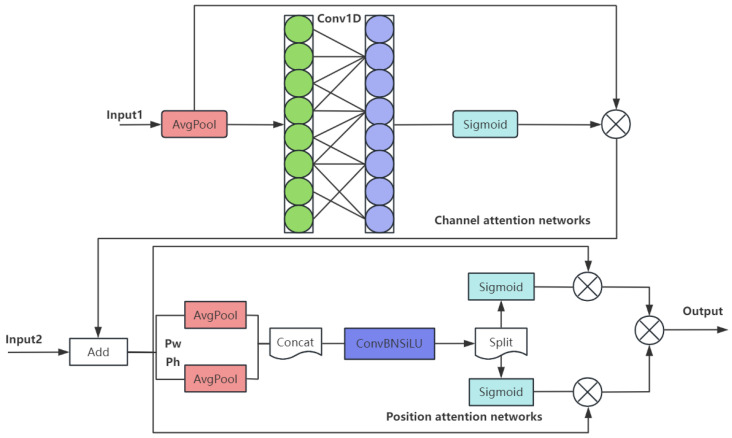
The structure of the CPAM module.

The feature information of SSFF and TFE modules is integrated to focus on the channel and spatial location related to small objects, thereby improving the detection and segmentation performance, as shown in Figure 5, which contains channel and location attention networks; w and h denote width and height respectively, ⊗ denotes the run of Hadamard product.

Computational formula:

(21) $$C=\psi \left(k\right)=2^{\left(\gamma \times k-b\right)}$$

(22) $$k=\psi \left(C\right)={\left|\left.\frac{{log}_{2}\left(C\right)+b}{\gamma }\right|\right.}_{odd}$$

Here, *γ* and *b* are scaling parameters that control the ratio of the convolution kernel size *k* to the channel dimension *C*.

**Figure 6:**
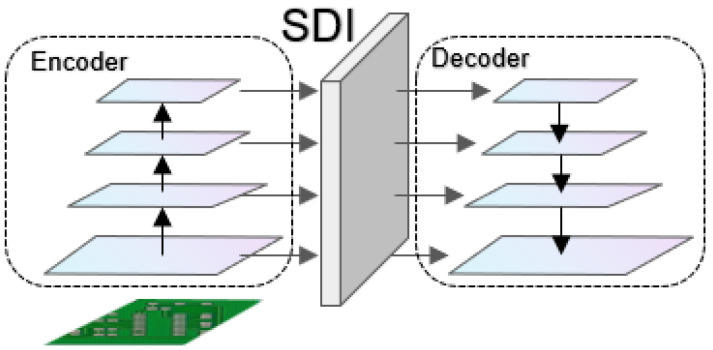
The overall architecture of the U-Net v2 model consists of the encoder, the SDI (semantic and detail injection) module, and the decoder.

Multi-level feature fusion module SDI is discussed in [33]. U-Net v2 is a modified U-Net architecture that aims to enhance the performance of image segmentation by redesigning skip connections. The proposed model improves the quality of feature maps by injecting semantic information and detailed information into the multi-level feature maps generated by the encoder, as shown in Figure 6.

1. Spatial and channel attention mechanism

Enhance the ability of feature maps to integrate local spatial information and global channel information.

(23) $$f_{i}^{1}=\phi_{i}^{c}\left(\varphi_{i}^{s}\left(f_{i}^{0}\right)\right)$$

Here, $f_{i}^{1}$ is the original feature map at level *i*; $\varphi_{i}^{s}$ and $\phi_{i}^{c}$ represent the parameters of the spatial and channel attention mechanism, respectively.

2. Semantic and detail injection SDI module

High-level features (semantic information) and low-level features (detail information) are fused into each level feature map by Hadamard product. Feature map adjustment and fusion:

(24) $$f_{ij}^{3}\left\{\begin{array}{c} D\left(f_{j}^{2},\left(H_{i},W_{i}\right)\right)\;if\;j<i,\\ I\left(f_{j}^{2}\right)\;\;\;\;if\;j=i,\\ U\left(f_{j}^{2},\left(H_{i},W_{i}\right)\right)\;if\;j>i, \end{array}\right.$$

Here, *D*, *I*, and *U* represent adaptive average pooling, identity mapping, and bilinear interpolation, respectively.

3. Smooth convolution

Smooth the adjusted feature map by 3 × 3 convolution:

(25) $$f_{ij}^{4}=\theta_{ij}\left(f_{ij}^{3}\right)$$

Here, $\theta^{ij}$ represents the parameters of the smoothed convolution.

### 2.3. Synergistic Mechanism Analysis

The YOLO-SUMAS model proposed in this paper constructs a closed-loop framework of “feature enhancement—lightweight inference—multi-scale fusion—loss optimization” through the collaborative optimization of multiple modules (Figure 1), and all the modules complement each other:

1. SCSA dynamically focuses the local details (such as missing solder joints) and global semantics (such as short circuit areas) of PCB defects through spatial-channel co-attention. Its progressive channel compression strategy reduces redundant computations and provides highly discriminative features for subsequent modules. As well, the optimized features of SCSA output are lightweight processed by the UIB module of MobileNetV4 (Figure 3) to reduce the number of parameters while retaining key information.
2. The UIB module of MobileNetV4 combines with the inverted bottleneck structure to achieve efficient multi-scale feature extraction on the mobile terminal. Its MobileMQA attention reduces the amount of computation through spatial downsampling, and forms a two-stage optimization of “coarse sift-fining” with SCSA. In addition, the features of MobileNetV4 after lightweight are input into ASF-SDI Neck, and the small target details are enhanced by weighted bidirectional pyramid to avoid information loss caused by lightweight.
3. Through the semantic-detail injection module, ASF-SDI fuses high-level semantics (such as defect categories) with low-level geometric features (such as edge shapes) to solve the problem of large differences in PCB defect sizes. In addition, after multi-scale feature optimization, ASF-SDI and Unified-IoU refine the localization by dynamic bounding box scaling to reduce missed detection of overlapping defects.
4. Unified-IoU introduces a bidirectional weight allocation strategy, which imposes higher loss weights on low confidence prediction boxes (difficult samples), and uses cosine decay to balance training speed and accuracy. The loss optimization results of Unified-IoU reverse guide the feature extraction priorities of SCSA and MobileNetV4, forming an end-to-end optimization and closed-loop feedback to the overall framework.

### 2.4. Implementation Details

Under the framework of YOLOv8, we modify the code of backbone and neck, and then add the above improved modules and interact with each other to play a collaborative optimization, enhance the model training ability, and build a scientific and efficient closed-loop framework.

## 3. Experimental Results and Discussion

### 3.1. Dataset

**Figure 7:**
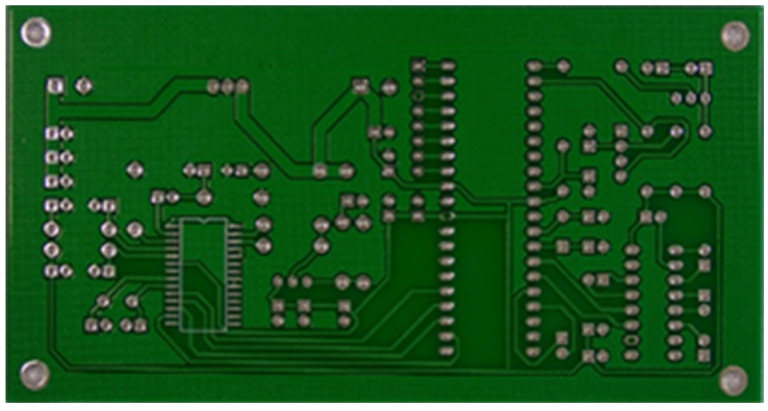
Dataset example.

**Figure 8:**
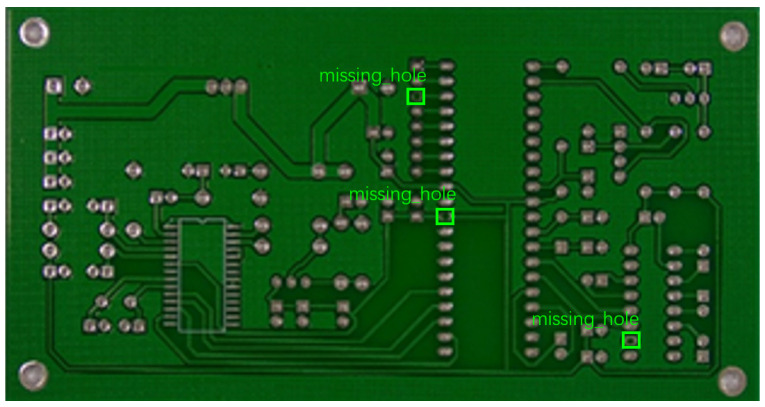
Example of dataset defect information (missing holes).

The image dataset used for model training and detection in the experiments comes from the PKU-Market-PCB dataset released by the Open Laboratory for Intelligent Robotics at Peking University, which contains a total of 693 PCB images for six kinds of defects (missing holes, mouse bites, open circuits, short circuits, spur, and spurious copper). Considering the small number of sample images and defects available in the original dataset, direct use for training may not produce satisfactory results due to insufficient data volume. In addition, the PCB images in the dataset are well-shaped rectangles with a minimum tilt angle. In practice, if the PCBs are placed at an inclined angle or subjected to other disturbances, the detection results may be adversely affected. Therefore, to strengthen the generalization ability of the network [34] and considering the symmetric nature of PCB layout, image enhancement—including mosaic enhancement, flipping, random noise addition, and brightness adjustment—was used to randomly extend the dataset and improve the generalization ability of the detection model to 10,668 images. Figure 7 and Figure 8 represent an example dataset.

**Figure 9:**
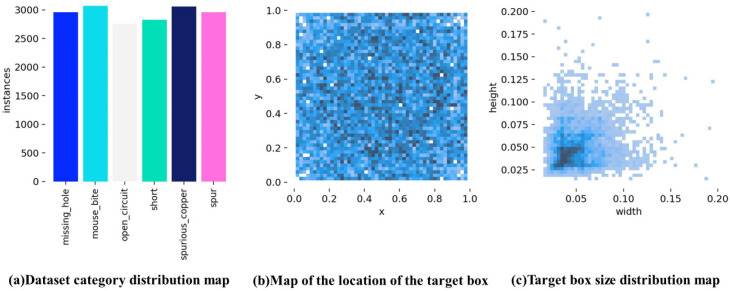
Enhanced defect distribution information.

To visualize the defect information after data enhancement, we analyzed the overall distribution, size, and number of defects, as shown in Figure 9. As shown in Figure 9a, the enhanced dataset generated by random data enhancement has different numbers of images for each of the six defect categories, but the differences are relatively small. Figure 9b shows the location of the centroid of all the defect detection bounding boxes in the enhanced images. It is apparent from Figure 9b that the centroids of all enhanced defects are widely dispersed, indicating that the defects do not follow a clear spatial pattern. Figure 9c shows the width and height of all bounding boxes in the enhanced dataset. As shown in Figure 9c, most of the bounding boxes are centered near the origin, and their dimensions are mainly in the range of [0.025, 0.05]. This indicates that most of the detected bounding boxes are small, and most defects are small-sized targets.

#### 3.1.1. Data Set Division

According to the size of the PCB defective circuit board data set, the division ratio of the training set, validation set, and test set is set to be about 8:1:1. A total of 8640 pictures are set as the training set, 961 pictures as the validation set, and 1067 pictures as the test set. To ensure the reliability and fairness of the experiment, by randomly disrupting the dataset while dividing the dataset, the sequentiality and correlation of the data can be eliminated, and the model is less affected by the order in which the data are arranged.

#### 3.1.2. Conversion of the Dataset

The format of the YOLO dataset is stored in the form of a text file, and the labeling file in the PCB dataset is stored in XML format. Therefore, before training with the YOLO model, the label files in XML format are converted to the text file format required by YOLO so that the model can correctly read and parse the information required for the target detection task.

### 3.2. Experimental Platform

#### 3.2.1. Experiment Configuration

Our experimental configuration is shown in Table 1 below.

#### 3.2.2. Experimental Parameters for This Experiment

For this experiment, the training parameters are set as follows: πre-trained weights are loaded; the training device is GPU, and the input image size is 640 × 640 pixels. The data loader only uses the main thread for loading, i.e., workers are set to 0. The random seed value seed is set to 0. Mixed-precision amp training is set to false. The learning rate was set to 0.01 to find a balance between training stability and convergence speed, with a small learning rate leading to slow convergence and a large learning rate leading to mAP oscillations during training. The batch size is set to 8, which provides the best balance between GPU memory usage and model generalization performance. The momentum is set to 0.937, which accelerates convergence by maintaining a stable gradient flow. Weight decay is set to 0.0005, and model regularization during training prevents overfitting. The number of training rounds is set to 100, and this number of training epochs is sufficient for the model to achieve the best performance without overfitting, as observed by the learning curve.

#### 3.2.3. Experimental Evaluation Metrics

The experiments use the intersection and integration ratio (IOU), Precision, Recall, mean average precision (mAP), F1 value, Params, and Gigafloating point operations per second (GFLOPs) as the key performance evaluation metrics. The *IOU* refers to the ratio of area overlap between the obtained detection results and the true value frame, which can be denoted as

(26) $$IOU=\frac{A\cap B}{A\cup B}$$

Precision is the proportion of true instances (true to positive category) among all samples classified as positive instances (positive category). It measures the risk of the classifier misclassifying negative examples (negative categories) as positive examples. The formula for the precision rate (Precision Rate = True Examples/True Examples + False Positive Examples) can be expressed as follows:

(27) $$P=\frac{TP}{TP+FP}$$

In the formula, *TP* (True Example) is the prediction of a positive example, and if the true result is positive, the prediction is correct. *TN* (False Positive Example) is the prediction of a positive example, and the true result is negative, so the prediction is incorrect.

Recall is the proportion of positive cases that are classified out of all samples that are true positive cases. It measures the ability of the classifier to correctly detect positive examples. Recall is calculated as true examples/(true examples + false negative examples):

(28) $$R=\frac{TP}{TP+FN}$$

In the formula *FP* (False Negative Example), if the prediction result is negative and the true result is positive, the prediction is wrong. *FN* (True Negative Example) is the prediction result is negative, and the true result is negative, so the prediction is correct.

*F*1 value is the index that combines the precision and recall:

(29) $$F1=\frac{2\times \left(P\times R\right)}{P+R}$$

Compared to *F*1 values, Mean Average Precision Mean (*mAP*) synthesizes performance across all categories and provides a more comprehensive assessment of the model’s accuracy and recall on the target detection task.

(30) $$mAP=\frac{1}{N}\sum_{i=1}^{N}APi$$

Params and GFLOPs represent the parameter size and computational complexity of the model, respectively, which are key indicators of model complexity and resource requirements. In addition, the FPS value of frames per second is used to evaluate the inference speed of the model, indicating how many images the model can process per second. Higher FPS values reflect the model’s ability to handle real-time detection tasks.

### 3.3. Experimental Results

#### 3.3.1. Comparison Experiments

Compare the performance of SCSA enhancement through comparison experiments, in which CBAM, CoordAtt, ECA, GAM, and SCSA modules were added to the YOLOv8 model in turn to compute P-precision, Recall, map@50, Params, and GFLOPs.

Based on the data in Table 2, we compared and analyzed the performance of different detection methods. Five detection methods are listed in the table: CBAM, CoordAtt, ECA, GAM, and SCSA. In terms of precision (P), the SCSA method performs the best at 97.2%, followed by ECA and CBAM. In terms of recall (Recall), the ECA method is the highest at 95.2%, while SCSA follows closely. In terms of average precision (mAP@50), SCSA also leads with 97.5%, followed by ECA and CoordAtt. In terms of model complexity, the number of parameters (Params) and the amount of computation (GFLOPs) are the two key metrics.

The GAM method has the highest number of parameters at 4.65 M, while the number of parameters for CoordAtt and ECA is the lowest, both at 3.01 M. In terms of computation volume, GAM is also the highest, 9.4 G. Taken together, the SCSA method excels in precision, recall, and average precision, and strikes a better balance between performance and efficiency, although its number of parameters and computation volume are slightly higher than that of CoordAtt and ECA, but considering its performance advantages, these additional resource consumptions are reasonable.

#### 3.3.2. Comparison Results

Table 3 shows the results of the six different types of PCB defect detection, demonstrating the performance comparison of the YOLOv8 model before and after it has been improved, i.e., the YOLO-SUMAS model, in the PCB defect detection task.

The YOLO-SUMAS model achieves significant optimization in several key performance metrics. First, the precision (P%) of the YOLO-SUMAS model is improved from 97.6% to 98.8%, and the recall (Recall%) is increased from 90% to 99.2%, i.e., the improved model is more accurate and comprehensive in identifying PCB defects. In addition, the frame rate (FPS) of the model is improved from 338.96 to 383.46, and the processing speed is enhanced, which is more favorable for application scenarios that require real-time processing. In terms of the mean accuracy (mAP@50), YOLO-SUMAS reaches 99.1%, which is a significant improvement compared to 94.4% of YOLOv8, indicating the enhancement of the overall detection performance of the model. Specifically, for each category of defects, YOLO-SUMAS has a lower average accuracy in open-circuit detection, but the performance difference is not significant. mAP@50 values are higher than those of YOLOv8 in the categories of short-circuits, Spurious copper, missing holes, rat bites, and spurs, with the most significant improvement in missing holes and spurs, which have been increased from 98.8% and 86.7% to 99.2% and 99.4%, respectively. The YOLO-SUMAS model improves the accuracy and efficiency when dealing with PCB defect detection tasks and also enhances the model’s ability to recognize different types of defects, which provides more reliable technical support in practical industrial inspection applications.

#### 3.3.3. Performance Data Visualization

The following comparison of prediction results is shown in the figure, where it can be seen that the model YOLO-SUMAS accuracy is overall better than the baseline model YOLOv8 accuracy.

**Figure 10:**
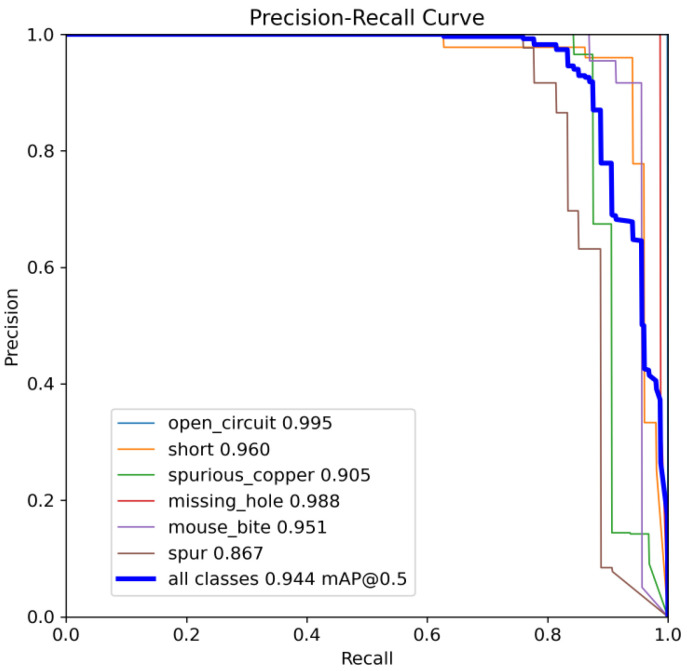
YOLOv8n accuracy–recall curve mAP@0.5.

**Figure 11:**
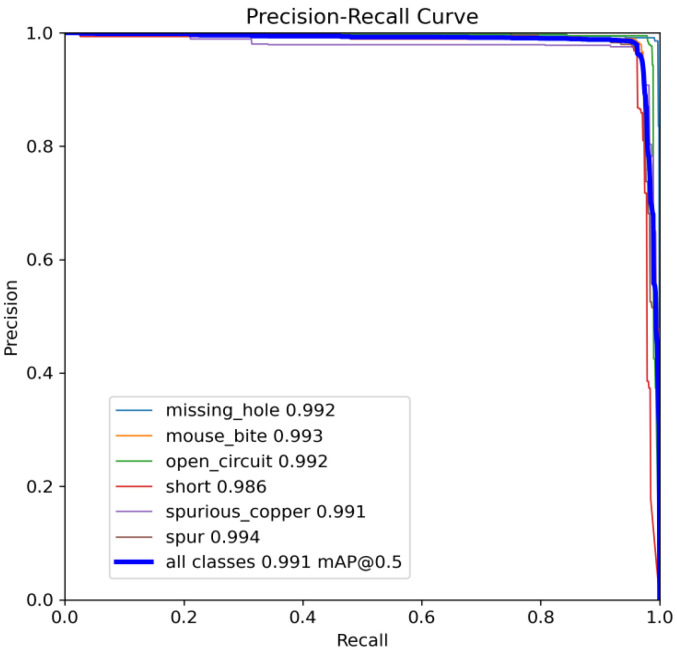
YOLO-SUMAS accuracy–recall curve mAP@0.5.

Figure 10 and Figure 11 show the Precision–Recall curves for the YOLOv8 and YOLO-SUMAS models for different defect detection categories, along with the corresponding average precision (mAP@0.5) values. As shown in Figure 10, the YOLOv8 model shows high precision and recall in detecting defects such as open circuits, short circuits, spurious copper, missing holes, and mouse bites. Among them, the detection performance of open and missing holes is particularly outstanding, with precision and recall close to or over 0.98. However, for the detection of spurious copper and mouse bites, the performance of YOLOv8 is relatively weak, with a precision and recall of 0.905 and 0.951, respectively. In contrast, the YOLO-SUMAS model in Figure 11 shows higher performance in all types of defect detection tasks. The precision and recall of all defect categories are close to or over 0.98, especially for the detection of short circuits, spurious copper, and mouse bites; the precision and recall of YOLO-SUMAS reach 0.986, 0.991, and 0.993, respectively.

According to Table 4, we compare and analyze the performance of YOLOv8 and YOLO-SUMAS in detecting PCB defects. Figures a to j show the comparison of defect types between the baseline model and the improved model, respectively, and it can be seen that the defect detection points are uniformly distributed and the confidence level is high. In the baseline model, the confidence level of the six defect detections is mainly located in 0.7 to 0.8, while the confidence level of the improved model for the six defect detections is mainly concentrated in 0.8 to 0.9, which is an improvement of about 10%. Figures m to x show the comparison of the heat maps of the baseline model and the improved model, respectively, and it can be seen that the distribution of the heat of the baseline model is relatively broad compared to that of the YOLO-SUMAS model, which is not as concentrated as the YOLO-SUMAS model. In summary, YOLO-SUMAS shows high accuracy and stability in detecting PCB defects and can provide high-quality results both in the detection of structural defects (e.g., open circuits, missing holes, and spurs) and functional defects (e.g., short circuits, spurious copper, and mouse bites).

#### 3.3.4. Optimization of Module Ablation Experiment

**Figure 12:**
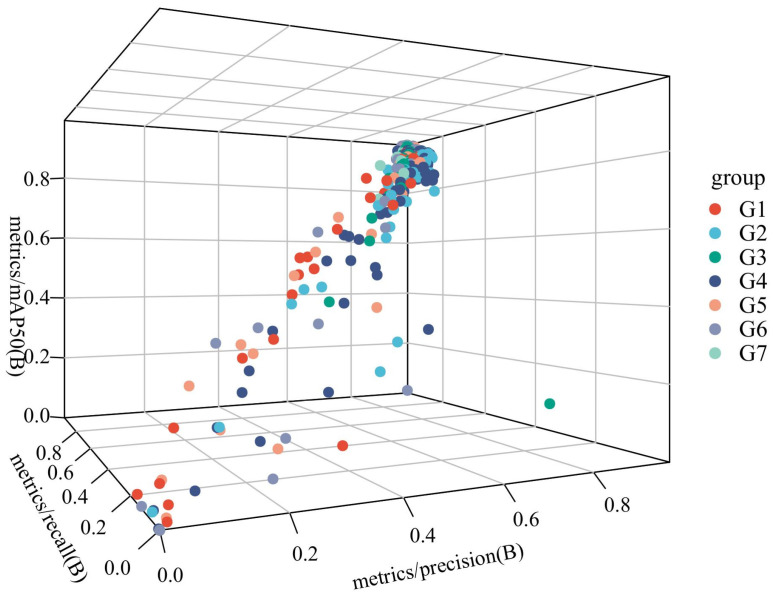
3D scatter plot.

Ablation experiments were performed to evaluate the contribution of the four modules to improved model performance. SCSA, Uni-IOU, MobileNetV4, and ASF-SDI are added to the model in this article in turn. The experimental design is shown in Table 5, while the 3D scatter plot of the experimental data is shown in Figure 12, where G1 to G9 denote Experiment 1 to Experiment 7, respectively:

Experiment 1: SCSA;

Experiment 2: Unified-IoU;

Experiment 3: MobileNetV4;

Experiment 4: ASF-SDI;

Experiment 5: SCSA and Unified-IoU;

Experiment 6: SCSA, Unified-IoU, and MobileNetV4;

Experiment 7: SCSA, Unified-IoU, MobileNetV4, and ASF-SDI.

1. Independent Module Contribution Analysis

SCSA enhances the model’s ability to focus on key semantic features, dynamically adjusts feature weights through the channel and spatial attention, and suppresses irrelevant noise; further, its mAP@50 value verifies its advantage in target localization accuracy. Unified-IoU is less accurate than SCSA when used alone and has limited improvement in boundary regression when applied independently as a loss-function optimization module, but its accuracy is close to SCSA’s. In terms of reducing false detections, it relies on other modules to provide high-quality feature inputs. MobileNetV4 has the highest recall rate among all modules, and its Universal Inverted Bottleneck structure efficiently extracts multi-scale features to improve small target detection. ASF-SDI cross-scale feature fusion strategy introduces redundancy information or gradient information when there is a lack of attention or efficient backbone support. It introduces redundant information or gradient conflicts, and the accuracy is relatively low.

2. The analysis of the module synergistic effect

The accuracy rate is improved to 98.2% in Experiment V. The attention mechanism of SCSA complements the boundary optimization of Unified-IoU; the former enhances the feature discriminative properties, and the latter refines the localization accuracy. After MobileNetV4 is added based on Experiment V, both recall and map@50 are greatly improved. The Universal Inverted Bottomed V4 structure of MobileNetV4 and the MobileMQA module efficiently deliver multi-level features to make up for SCSA’s shortcomings in high-level semantic fusion and form a strong synergy with it, while Unified-IoU optimization lightens the localization deviation of the backbone. Precision, recall, and mAP@50 peak in Experiment 7. SCSA enhances local feature discrimination and provides a high-quality feature base for other modules; MobileNetV4 provides efficient multi-scale characterization; ASF-SDI fuses details and semantics through cross-layer feature products in the complete framework; and Unified-IoU refines the loss computation. The four form a closed loop of “feature extraction–attention focusing–multi-scale fusion-loss optimization” to achieve a comprehensive performance breakthrough.

**Figure 13:**
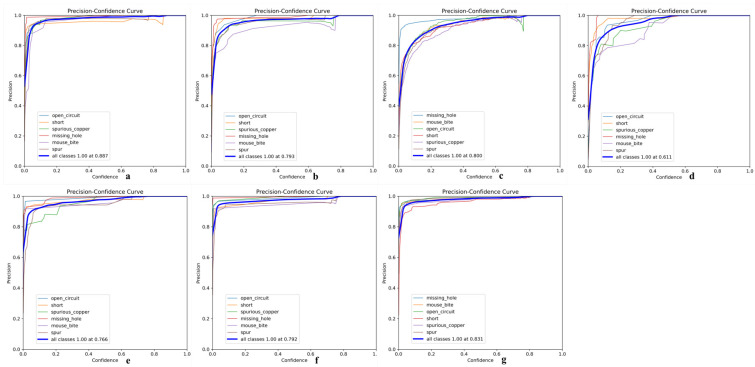
(**a**) Precision-confidence curve of Experiment 1; (**b**) Precision-confidence curve of Experiment 2; (**c**) Precision-confidence curve of Experiment 3; (**d**) Precision-confidence curve of Experiment 4; (**e**) Precision-confidence curve of Experiment 5; (**f**) Precision-confidence curve of Experiment 6; (**g**) Precision-confidence curve of Experiment 7.

**Figure 14:**
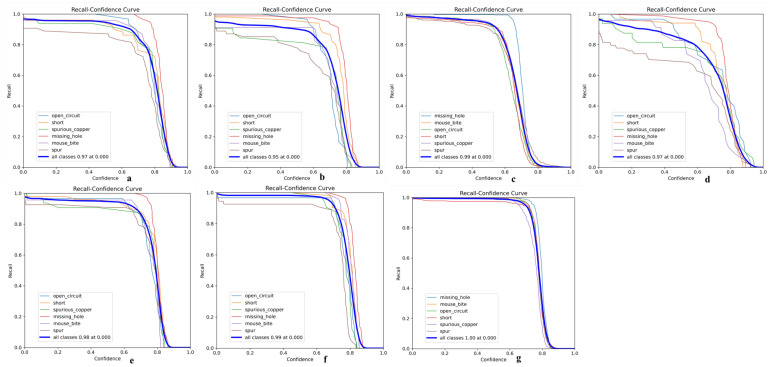
(**a**) Recall-confidence curve of Experiment 1; (**b**) Recall–confidence curve of Experiment 2; (**c**) Recall–confidence curve of Experiment 3; (**d**) Recall–confidence curve of Experiment 4; (**e**) Recall–confidence curve of Experiment 5; (**f**) Recall–confidence curve of Experiment 6; (**g**) Recall–confidence curve of Experiment 7.

**Figure 15:**
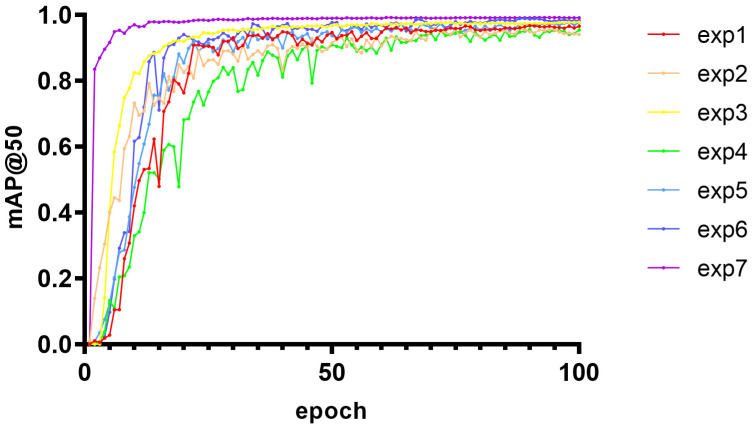
The variation trend of mAP@0.5 with training cycle under seven model configurations.

Its precision-confidence curve (Figure 13), recall rate-confidence curve (Figure 14), and mAP@50 curve (Figure 15) are as follows, where a–g in Figure 13 represents experiments I to VII; a–g in Figure 14 represents experiments I to VII; and exp1 to exp7 in Figure 15 represents experiments I to VII in tables.

#### 3.3.5. Comparison with Other Models

To verify the validity of the improved model, we base the YOLO-SSW against a wide range of object detection models’ Quasi-tests to extend our comparison to include Faster R-CNN [35], SSD [16], RTEDTR [36], Centernet [37], YOLOv3 [12], YOLOv5, YOLOv6 [14], YOLOv7 [15], YOLOv8n, YOLOv9 [38], YOLOv10n [39], and YOLO11 [40]; corresponding test results are shown in Table 6.

**Figure 16:**
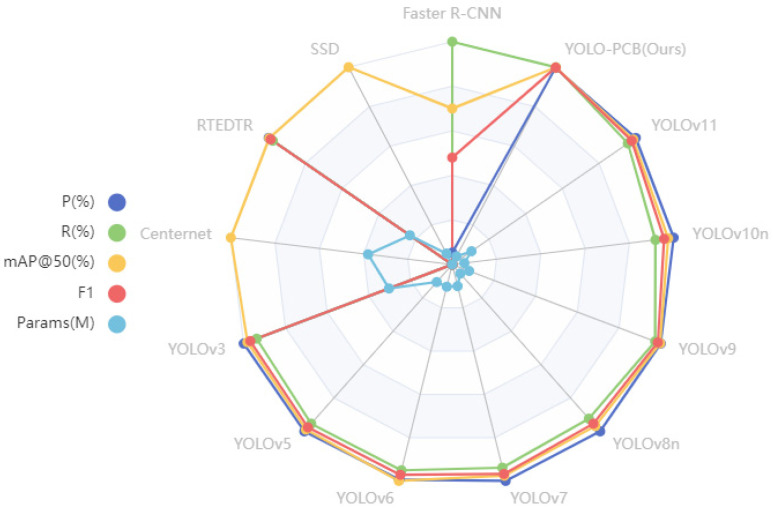
Performance comparison radar chart.

In terms of parameter scale, YOLOV10n has the smallest parameter (2.27 M), YOLOV3 has the largest parameter (61.55 M), and YOLO-SUMAS has a parameter of 3.33 M, which is at a low level. In terms of detection performance, YOLO-SUMAS’s mAP@50 reaches 99.1%, which is significantly higher than that of YOLOV9 (97.8%) and YOLOV5 (97.5%). Its precision (98.8%) is close to that of YOLOV11 (98.9%), but its recall rate (99.2%) is significantly higher than that of the latter (94.6%). In terms of the F1 score, YOLO-SUMAS leads with 99.0%, which is better than the highest value of 96.7% of other models. Although the F1 score of RTEDTR is 95.69%, the number of parameters (19.88 M) is about 6 times that of YOLO-SUMAS. Overall, YOLO-SUMAS has outstanding performance in the balance between parameter quantity and detection performance, especially in the recall rate and comprehensive indicators. The radar plot of the above performance comparison is shown in Figure 16.

## 4. Visualization Deployment

**Figure 17:**
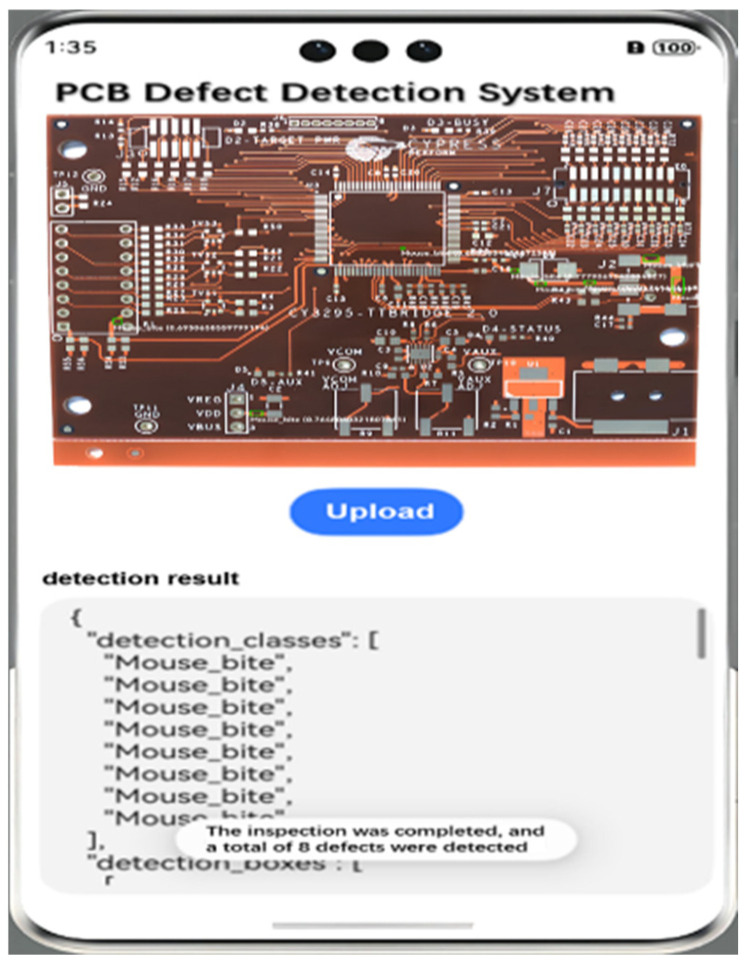
Mobile deployment interface.

We realize PCB board image uploading, target defect identification, and quantity detection by building the algorithm model deployment on the cloud server and making the design APP for calling, which is easy and convenient to operate, and the accuracy is scientific and reasonable, which is conducive to the visualization of quality inspection. The main function of the front-end interface is to provide an interface for uploading images and displaying the result information of network prediction. The local PCB image can be uploaded by clicking the upload image button on the page. At this time, the interface will pop up a pop-up window of AI prediction; please wait to reserve time for the execution of the back-end program. The back-end program receives the JSON data from the front-end at this time and will be converted into a trained network model for prediction; successful prediction of the image will be displayed in the detection results, and the network will be the category of defects, the target detection frame, and the confidence index, which is presented in the visual interface; the detection system will also be the prediction of the results of the MySql database to save the record. As shown in Figure 17, the network predicts a PCB image with 8 rat-bite type defects, and the indicators predicted by the network can be seen on the page.

## 5. Conclusions

Aiming at the accuracy and efficiency requirements of defect detection in high-density manufacturing scenarios of printed circuit boards (PCBs), this paper proposes an improved inspection model, YOLO-SUMAS, based on YOLOv8n. By integrating the SCSA attention mechanism, the Unified-IoU loss function, the MobileNetV4 backbone architecture, and the ASF-SDINeck structure, the model achieves a significant improvement in feature extraction, localization optimization, computational efficiency, and multi-scale fusion capability. Compared with traditional methods, the model validates the advantages of single-stage models in industrial real-time inspection with lower parameters and higher FPS while avoiding the high computational overhead of transformer-class methods. In a high-density PCB manufacturing scenario, the model demonstrates robustness in noise interference and uneven exposure environments through data augmentation, and its lightweight feature supports edge device deployment, reducing the hardware dependence and labor cost of traditional inspection means. Future research can focus on noise robustness enhancement (e.g., adversarial training), multimodal data fusion (combined with thermal imaging or electrical inspection), adaptive feature pyramid network optimization, and industrial deployment of model compression techniques (quantization and pruning) to further extend its potential application in cross-domain inspection tasks such as semiconductor packaging.

The results show that compared with the baseline model YOLOv8n, the YOLO-SUMAS model improves the precision, recall rate, mAP@50, and other key indicators by 1.2%, 9.2%, and 4.7%, respectively, while maintaining a high FPS, which verifies the effectiveness of the relevant improvements. Future work can focus on further optimizing the attention mechanism and exploring more sophisticated feature fusion methods to continue to improve the robustness and generalization ability of the model in noisy interference scenarios and eventually extend its applicability to defect detection tasks in other domains.

## Figures and Tables

**Table 1:** Parameter configuration.

Configure	Version
CPU	16 vCPU Intel(R) Xeon(R) Platinum 8481C
GPU	RTX 4090D (24 GB)
Python	3.8 (ubuntu20.04)
PyTorch	2.0.0
Cuda	11.8

**Table 2:** Comparative test result.

Classes	P (%)	Recall (%)	mAP@50 (%)	Params (M)	GFLOPs
CBAM	96.8	94	96.8	3.07	8.1
CoordAtt	96.4	94.6	97.2	3.01	8.1
ECA	96.6	95.2	97.4	3.01	8.1
GAM	96.3	94.1	96.7	4.65	9.4
SCSA	97.2	94.7	97.5	3.04	8.2

**Table 3:** Comparison of data before and after model improvement.

	P (%)	R (%)	FPS	F1	Params (M)	GFLOPs	mAP@50 (%)	AP@50(%)					
								Open Circuit	Short Circuits	Spurious Copper	Missing Hole	Mouse Bite	Spur
YOLOv8n	97.6	90	338.96	0.93	3.01	8.1	94.4	99.5	96	90.5	98.8	95.1	86.7
YOLO-SUMAS	98.8	99.2	383.46	0.99	3.04	8.2	99.1	99.2	98.6	99.1	99.2	99.3	99.4

**Table 4:** Prediction contrast chart.

YOLOv8	YOLO-SUMAS (Ours)	YOLOv8	YOLO-SUMAS (Ours)
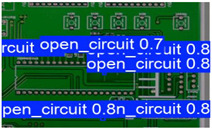 a	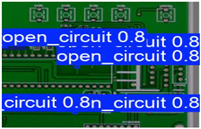 b	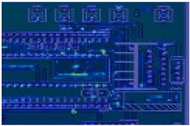 m	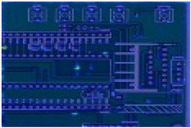 n
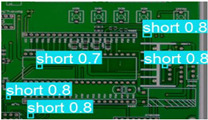 c	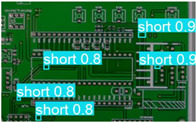 d	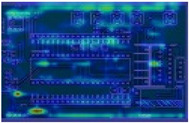 o	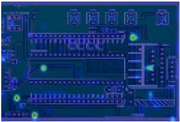 p
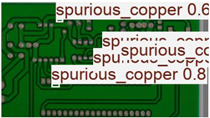 e	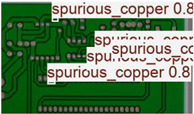 f	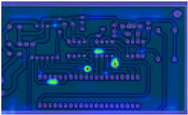 q	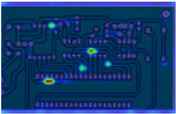 r
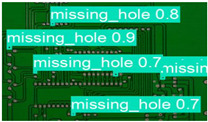 g	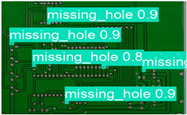 h	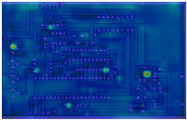 s	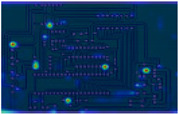 t
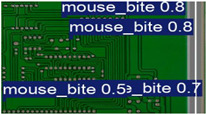 i	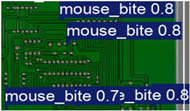 j	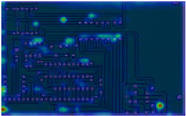 u	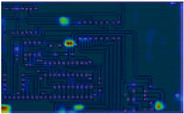 v
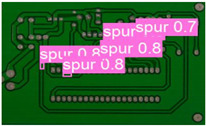 k	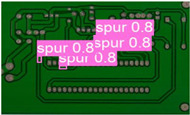 l	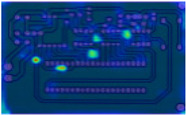 w	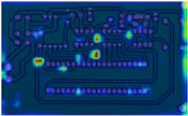 x

**Table 5:** Ablation data.

	SCSA	Unified-IoU	MobileNetV4	ASF-SDI	P (%)	Recall (%)	mAP50 (%)	F1
Exp1	✓	✕	✕	✕	97.2	94.7	97.5	0.96
Exp2	✕	✓	✕	✕	97	92.5	94.5	0.95
Exp3	✕	✕	✓	✕	96.6	95.5	97.7	0.96
Exp4	✕	✕	✕	✓	96.7	88.8	94.9	0.92
Exp5	✓	✓	✕	✕	98.2	94.4	97.6	0.96
Exp6	✓	✓	✓	✕	97.9	98.1	98.7	0.98
Exp7	✓	✓	✓	✓	98.8	99.2	99.1	0.99

**Table 6:** Performance comparison of different models.

Classes	P (%)	R (%)	mAP@50 (%)	F1	Params (M)
Faster R-CNN [35]	61.4	75.6	71.1	67.8	-
SSD [16]	-	-	72.3	-	25.1
RTEDTR [36]	96.8	94.6	96.5	95.69	19.88
Centernet [37]	-	-	94.69	-	32.7
YOLOv3 [12]	96.4	93.3	95.5	94.82	61.55
YOLOv5	98.6	94.0	97.5	96.2	9.1
YOLOv6 [14]	93.4	89.10	94.00	91.2	4.23
YOLOv7 [15]	96.0	89.9	93.6	92.8	6.0
YOLOv8n	97.6	90	94.4	93.0	3.01
YOLOv9 [38]	98.1	95.4	97.8	96.7	6.2
YOLOv10n [39]	96.8	88.6	94.3	92.52	2.27
YOLOv11 [40]	98.9	94.6	97.6	96.7	9.4
YOLO-SUMAS (Ours)	98.8	99.2	99.1	99.0	3.33

## Data Availability

Data are contained within the article.

## References

[1] Jiang W., Li T., Zhang S., Chen W., Yang J. (2023). PCB defects target detection combining multi-scale and attention mechanism. Eng. Appl. Artif. Intell..

[2] Zhou Y., Yuan M., Zhang J., Ding G., Qin S. (2023). Review of vision-based defect detection research and its perspectives for printed circuit board. J. Manuf. Syst..

[3] Zhang H., Jiang L., Li C. (2021). CS-ResNet: Cost-sensitive residual convolutional neural network for PCB cosmetic defect detection. Expert Syst. Appl..

[4] Cong S., Shang Z., Huang Q. (2022). Detection for printed circuit boards (PCBs) delamination defects using optical/thermal fusion imaging technique. Infrared Phys. Technol..

[5] Wang F., Zhou Y., Zhang X., Li Z., Weng J., Qiang G., Chen M., Wang Y., Yue H., Liu J. (2023). Laser-induced thermography: An effective detection approach for multiple-type defects of printed circuit boards (PCBs) multilayer complex structure. Measurement.

[6] El Belghiti Alaoui N., Cassou A., Tounsi P., Boyer A., Viard A. (2019). Using infrared thermal responses for PCBA production tests: Feasibility study. Microelectron. Reliab..

[7] Girshick R., Donahue J., Darrell T., Malik J. Rich feature hierarchies for accurate object detection and semantic segmentation. Proceedings of the IEEE Conference on Computer Vision and Pattern Recognition.

[8] He K., Zhang X., Ren S., Sun J. (2015). Spatial pyramid pooling in deep convolutional networks for visual recognition. IEEE Trans. Pattern Anal. Mach. Intell..

[9] Girshick R. (2015). Fast r-cnn. Proceedings of the IEEE International Conference on Computer Vision.

[10] Ren S. (2015). Faster r-cnn: Towards real-time object detection with region proposal networks. arXiv.

[11] Redmon J., Farhadi A. YOLO9000: Better, faster, stronger. Proceedings of the IEEE Conference on Computer Vision and Pattern Recognition.

[12] Redmon J. (2018). Yolov3: An incremental improvement. arXiv.

[13] Bochkovskiy A., Wang C.-Y., Liao H.-Y.M. (2020). Yolov4: Optimal speed and accuracy of object detection. arXiv.

[14] Li C., Li L., Jiang H., Weng K., Geng Y., Li L., Ke Z., Li Q., Cheng M., Nie W. (2022). YOLOv6: A single-stage object detection framework for industrial applications. arXiv.

[15] Wang C.-Y., Bochkovskiy A., Liao H.-Y.M. YOLOv7: Trainable bag-of-freebies sets new state-of-the-art for real-time object detectors. Proceedings of the IEEE/CVF Conference on Computer Vision and Pattern Recognition.

[16] Liu W., Anguelov D., Erhan D., Szegedy C., Reed S., Fu C.-Y., Berg A.C. (2016). SSD: Single shot multibox detector. Proceedings of the Computer Vision–ECCV 2016: 14th European Conference.

[17] Tang S., He F., Huang X., Yang J. (2019). Online PCB defect detector on a new PCB defect dataset. arXiv.

[18] Li C.J., Qu Z., Wang S.Y., Bao K.H., Wang S.Y. (2021). A method of defect detection for focal hard samples PCB based on extended FPN model. IEEE Trans. Compon. Packag. Manuf. Technol..

[19] Ding R., Dai L., Li G., Liu H. (2019). TDD-net: A tiny defect detection network for printed circuit boards. CAAI Trans. Intell. Technol..

[20] Wu L., Zhang L., Zhou Q. (2022). Printed circuit board quality detection method integrating lightweight network and dual attention mechanism. IEEE Access.

[21] Tsai D.-M., Chou Y.-H. (2019). Fast and precise positioning in PCBs using deep neural network regression. IEEE Trans. Instrum. Meas..

[22] Chen W., Huang Z., Mu Q., Sun Y. (2022). PCB defect detection method based on transformer-YOLO. IEEE Access.

[23] Tang J., Liu S., Zhao D., Tang L., Zou W., Zheng B. (2023). PCB-YOLO: An improved detection algorithm of PCB surface defects based on YOLOv5. Sustainability.

[24] Annaby M., Fouda Y., Rushdi M.A. (2019). Improved normalized cross-correlation for defect detection in printed-circuit boards. IEEE Trans. Semicond. Manuf..

[25] Bhattacharya A., Cloutier S.G. (2022). End-to-end deep learning framework for printed circuit board manufacturing defect classification. Sci. Rep..

[26] Spadaro G., Vetrano G., Penna B., Serena A. Towards One-Shot PCB Component Detection with YOLO. Proceedings of the Image Analysis and Processing—ICIAP 2023 Workshops.

[27] Benjumea A., Teeti I., Cuzzolin F., Bradley A. (2021). YOLO-Z: Improving small object detection in YOLOv5 for autonomous vehicles. arXiv.

[28] Chen X., Wu Y., He X., Ming W. (2023). A comprehensive review of deep learning-based PCB defect detection. IEEE Access.

[29] Si Y., Xu H., Zhu X., Zhang W., Dong Y., Chen Y., Li H. (2024). SCSA: Exploring the synergistic effects between spatial and channel attention. arXiv.

[30] Luo X., Cai Z., Shao B., Wang Y. (2024). Unified-IoU: For High-Quality Object Detection. arXiv.

[31] Qin D., Leichner C., Delakis M., Fornoni M., Luo S., Yang F., Wang W., Banbury C., Ye C., Akin B. (2024). MobileNetV4: Universal models for the mobile ecosystem. Proceedings of the European Conference on Computer Vision.

[32] Kang M., Ting C.M., Ting F.F., Phan R.C.W. (2024). ASF-YOLO: A novel YOLO model with attentional scale sequence fusion for cell instance segmentation. Image Vis. Comput..

[33] Peng Y., Sonka M., Chen D.Z. (2023). U-net v2: Rethinking the skip connections of u-net for medical image segmentation. arXiv.

[34] Liu G., Wen H. (2021). Printed circuit board defect detection based on MobileNet-Yolo-Fast. J. Electron. Imaging.

[35] Li X., Zhang Z., Zhao P. (2024). Improvement of YOLOv8 Detection Algorithm for Worker-Related Objectives in Construction Scenarios. Electronic Engineering and Informatics.

[36] Zhao Y., Lv W., Xu S., Wei J., Wang G., Dang Q., Liu Y., Chen J. Detrs beat yolos on real-time object detection. Proceedings of the IEEE/CVF Conference on Computer Vision and Pattern Recognition.

[37] Wang J., Xie X., Liu G., Wu L. (2025). A Lightweight PCB Defect Detection Algorithm Based on Improved YOLOv8-PCB. Symmetry.

[38] Wang C.-Y., Yeh I.-H., Mark Liao H.-Y. (2024). Yolov9: Learning what you want to learn using programmable gradient information. Proceedings of the European Conference on Computer Vision.

[39] Wang A., Chen H., Liu L., Chen K., Lin Z., Han J. (2024). YOLOv10: Real-Time End-to-End Object Detection [J/OL]. arXiv.

[40] Khanam R., Hussain M. (2024). Yolov11: An overview of the key architectural enhancements. arXiv.

